# Matrix Metalloproteinases-7 and Kidney Fibrosis

**DOI:** 10.3389/fphys.2017.00021

**Published:** 2017-02-10

**Authors:** Ben Ke, Chuqiao Fan, Liping Yang, Xiangdong Fang

**Affiliations:** ^1^The Third Hospital of NanchangNanchang, China; ^2^Nanchang University School of MedicineNanchang, China; ^3^Department of Nephrology, The Second Affiliated Hospital to Nanchang UniversityNanchang, China; ^4^Department of Breast Surgery, Jiangxi Cancer HospitalNanchang, China

**Keywords:** MMP-7, epithelial-mesenchymal transition, extracellular matrix, TGF-β

## Abstract

Matrix metalloproteinase-7 (MMP-7) is a secreted zinc- and calcium-dependent endopeptidase that degrades a broad range of extracellular matrix substrates and additional substrates. MMP-7 playsa crucial role in a diverse array of cellular processes and appears to be a key regulator of fibrosis in several diseases, including pulmonary fibrosis, liver fibrosis, and cystic fibrosis. In particular, the relationship between MMP-7 and kidney fibrosis has attracted significant attention in recent years. Growing evidence indicates that MMP-7 plays an important role in the pathogenesis of kidney fibrosis. Here, we summarize the recent progress in the understanding of the role of MMP-7 in kidney fibrosis. In particular, we discuss how MMP-7 contributes to kidney fibrotic lesions via the following three pathways: epithelial-mesenchymal transition (EMT), transforming growth factor-beta (TGF-β) signaling, and extracellular matrix (ECM) deposition. Further dissection of the crosstalk among and regulation of these pathways will help clinicians and researchers develop effective therapeutic approaches for treating chronic kidney disease.

## Introduction

The prevalence of chronic kidney disease (CKD) in the United States is decreasing, but its incidence worldwide continues to increase (Fried and Palevsky, 2016; Zhang et al., 2016). Renal fibrosis, the common final outcome of a wide variety of progressive CKDs, is a complicated process characterized by increased fibroblast proliferation and extracellular matrix (ECM) accumulation, which lead to renal tubule fibrosis, glomerular sclerosis, renal artery stenosis, and chronic inflammatory cell infiltration (Liu, 2011). It is believed that the mechanism underlying renal fibrosis development is related to ECM accumulation caused by epithelial-mesenchymal transition (EMT), transforming growth factor-beta (TGF-β) signaling, chronic hypoxia, and oxidative stress (Liu, 2011). Unfortunately, there is no effective cure for renal fibrosis, and its progression to end-stage renal disease (ESRD) necessitates dialysis or kidney transplantation (Boor et al., 2010; Liu, 2011).

Metalloproteinase-7 (MMP-7) is a secreted zinc- and calcium-dependent endopeptidase that degrades a broad range of ECM substrates (Brabletz et al., 1999; Tan and Liu, 2012). MMP-7 is also known to cleave non-ECM proteins, such as E-cadherin and Fas ligand (FasL), and thus plays an important role in the regulation of diverse biological processes, such as EMT and cell apoptosis (Mitsiades et al., 2001; McGuire et al., 2003; Ii et al., 2006). MMP-7 is a downstream target gene of Wnt/β-catenin signaling and is involved in renal fibrosis via β-catenin signaling (Tan et al., 2014). Recent evidence suggests that MMP-7 is not only a biomarker for renal fibrosis but also a major pathogenic mediator in fibrotic lesion progression (Zhou et al., 2016). Mice with genetically ablated MMP-7 are largely protected against the development of renal fibrotic lesions after unilateral ureteral obstruction (UUO) and display much less collagen 1 and fibronectin accumulation and deposition in renal parenchyma, as well as reduced myofibroblast activation (Zhou et al., 2016). Furthermore, MMP-7 promotes renal fibrosis by inducing E-cadherin proteolytic degradation, ECM accumulation, and TGF-βsignaling activation (He et al., 2012; Oelusarz et al., 2013; Xiao et al., 2016). Pharmacological blockade of MMP-7 expression effectively attenuates renal injury and fibrosis (Xiao et al., 2016). Therefore, in this review, we aim to summarize recent findings regarding the role of MMP-7 in the pathogenesis of kidney fibrosis to facilitate the development of effective therapies for CKD.

## Biological characteristics of MMP-7

Matrix metalloproteinases (MMPs) are zinc-containing endopeptidases with a broad range of substrate specificities. They are secreted by keratinocytes and dermal fibroblasts in response to multiple stimuli, such as oxidative stress, UV radiation, and cytokines (Kim et al., 2011; Hwang et al., 2012; Ham et al., 2014). At least 28 different types of MMPs that play important roles in various pathophysiological processes, including aging, wound healing, skeletal growth, and remodeling, arthritis, inflammation, angiogenesis, and cancer (Jung et al., 2010; Sbardella et al., 2012), have been identified.

MMP-7, also known as matrilysin, matrilysin-1, and pump-punctuated metalloproteinase, is the smallest member of the MMP family (Muller et al., 1988; Woessner and Taplin, 1988; Quantin et al., 1989; Miyazaki et al., 1990; Abramson et al., 1995). MMP-7 is structurally different from the other members of the family because it lacks a C-terminal hemopexin domain (Gaire et al., 1994). MMP-7 is expressed at very low levels in adults and in only a few tissues but is upregulated in a variety of disease states, including cancer and idiopathic pulmonary fibrosis (Zuo et al., 2002; Huang et al., 2005; Ramankulov et al., 2008).

Increasing evidence suggests that MMP-7 plays an active role in renal pathology. MMP-7 contributes to renal tubular injury and tubulointerstitial fibrosis progression via Wnt 4 (Surendran et al., 2004). MMP-7 expression is not detected in healthy human renal tubular epithelium (Surendran et al., 2004). However, MMP-7 expression is upregulated in autosomal dominant polycystic kidney disease (Riera et al., 2006), renal biopsy samples from hydronephrotic patients with progressive disease requiring hemodialysis (Henger et al., 2004) and canine X-linked Alport syndrome (Rao et al., 2005). In addition, MMP-7 has been implicated in renal carcinoma development and progression (Patraki and Cardillo, 2007; Sarkissian et al., 2008). Moreover, MMP-7 is part of a group of genes that were significantly upregulated in very old kidneys exhibiting a higher rate of histopathological changes, including glomerulosclerosis, interstitial fibrosis and tubular atrophy (Melk et al., 2005). Consequently, significant effort has been expended to understand the molecular and cellular mechanisms by which MMP-7 contributes to kidney fibrosis. Below, we will discuss three main pathways that were recently identified as linking MMP-7 with kidney fibrosis.

### MMP-7 and EMT

During EMT, renal epithelial cells are transformed into mesenchymal cells. These mesenchymal cells ameliorate tissue damage, causing the accumulation of ECM and the production of myofibroblasts, which are key effectors in ECM synthesis and deposition. EMT is characterized by reduced cell-cell contact, reduced expression of epithelial markers such as E-cadherin, and increased expression of mesenchymal markers such as α-SMA, type I collagen, and fibronectin (Son and Moon, 2010). Although many studies have shown that EMT in the kidney may lead to renal fibrosis, the relationship between EMT and renal fibrosis remains controversial (Menon and Ross, 2016).

MMP-7 mediates tubular epithelial-interstitial fibroblast communication through Wnt/β-catenin signaling (Zhou et al., 2013, Figure 1), an evolutionarily conserved pathway that regulates cell fate, organ development, and tissue homeostasis, as well as injury and repair. Wnt/β-catenin signaling is relatively silent in the normal adult kidney but is activated after renal injury in a wide variety of animal models and human kidney disorders (Clevers and Nusse, 2012). Canonical Wnt signaling activation leads to β-catenin stabilization and nuclear translocation, followed by binding of β-cateninto T cell factor (TCF)/lymphoidenhancer–binding factor to stimulate the transcription of Wnt target genes, a common pathological occurrence in a wide variety of CKDs (Nelson et al., 2011). Tubular activation of β-catenin after injury leads to EMT and upregulates tubular expression and secretion of MMP-7 (Liu, 2010; He et al., 2012). As a result, secreted MMP-7 induces the expression of FasL, a key player in the extrinsic, receptor-mediated apoptosis pathway (Villa-Morales and Fernandez-Piqueras, 2012), in interstitial fibroblasts and potentiates apoptosis of these cells (Zhou et al., 2013). Loss of tubular β-catenin results in enhanced interstitial fibroblast survival due to decreased MMP-7 expression (Zhou et al., 2013).

**Figure 1:**
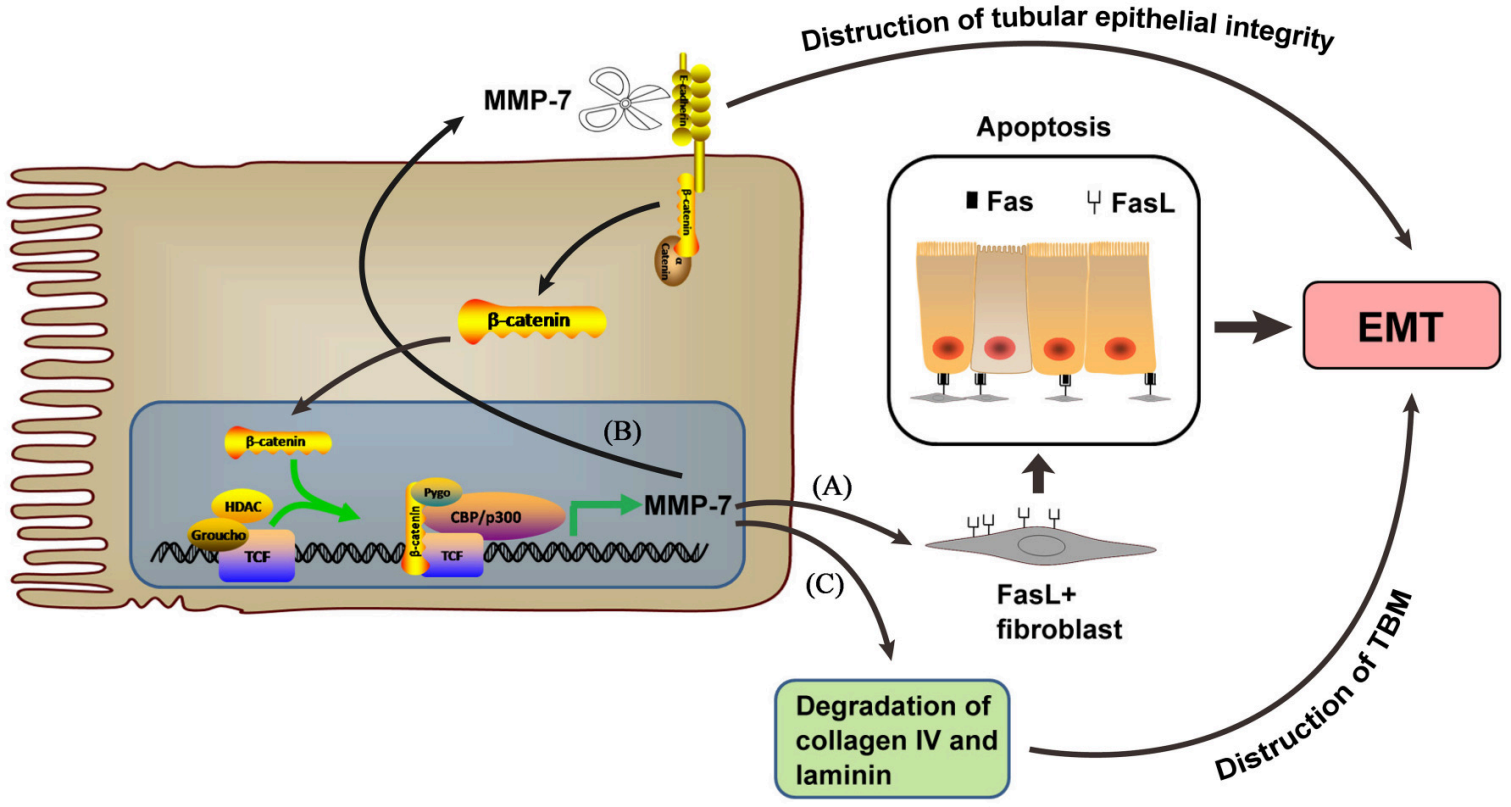
**MMP-7, which is expressed upon β-catenin activation, mediates EMT through the following 3 pathways. (A)** Inducing FasL expression in interstitial fibroblasts and potentiating their apoptosis. **(B)** Inducing tubular epithelial integrity destruction by regulating E-cadherin degradation. **(C)** Inducing TBM destruction by degrading collagen type IV and laminin.

MMP-7 induces E-cadherin proteolytic degradation and β-catenin signaling activation independent of Wnt to promote EMT (McGuire et al., 2003; Zhou et al., 2016). E-cadherin is an epithelial cell adhesion receptor essential for the maintenance of tubular epithelial integrity. E-cadherin destruction is the initial step during EMT and disrupts tubular epithelial cell integrity (Liu, 2004). Shibata et al. (2009) found that MMP-7 reduces E-cadherin levels and promotes EMT in diseased kidneys, contributing to tubular epithelial injury (Zhou et al., 2013). One consequence of MMP-7-mediated E-cadherin degradation is β-catenin liberation independent of Wnt (Zhou et al., 2016). The liberated β-catenin then translocates into the nucleus as a transcription regulator and drives the expression of target genes, including MMP-7(He et al., 2010; Tan and Liu, 2012). An MMP-7-knockout mouse model showed that MMP-7 is an important pathogenic mediator that triggers the activation of β-catenin signaling and promotes renal fibrosis (Zhou et al., 2016).

MMP-7 also degrades collagen type IV and laminin to induce EMT (Tan and Liu, 2012, Figure 1). MMP-7 cleaves not only elastin but also many other ECM substrates, such as collagen type IV and laminin (Sbardella et al., 2012), which are the major components of the tubular basement membrane (TBM). Increased MMP-7 aggravates TBM damage, which facilitates tubular EMT in fibrotic kidneys (Tan and Liu, 2012).

Advanced CKD is characterized by chronic hypoxia in the renal interstitium (Mimura and Nangaku, 2010). Hypoxia can lead to tubular EMT or apoptosis, activate resident fibroblasts and impair peritubular capillaries, creating a cycle of chronic hypoxia and resulting in renal fibrosis (Liu, 2011). MMP-7 is a major MMP activator in conditioned medium of hypoxic bladder smooth muscle cells (BSMCs) and is also transcriptionally induced at 6 h of hypoxia in an Erkl/2-dependent manner (Sabha et al., 2006). In addition, MMP-7 is upregulated in macrophages at sites of inflammation, such as wounds and tumors, in response to hypoxia (Crowther et al., 2001). The increase in the ratio of active (18 kDa) to inactive (28 kDa) MMP-7 coincides with the peak period of hypoxia-induced Erkl/2 activation. Moreover, hypoxia activates β-catenin in human macrophages by inducing MMP-7mRNA expression (Deguchi et al., 2009).

### MMP-7 and TGF-β signaling

TGF-β is a pleiotropic cytokine that plays an important role in cell proliferation and differentiation and induces the synthesis of ECM components. TGF-β is also a major cytokine/growth factor involved in renal fibrosis (Meng et al., 2012). Smad proteins are highly conserved transcription factors that mediate numerous effects of the TGF-β superfamily (Massague, 2012). TGF-β1 stimulates renal tubular EMT, which is a crucial process in tubulointerstitial fibrosis development (Carew et al., 2012).

**Figure 2:**
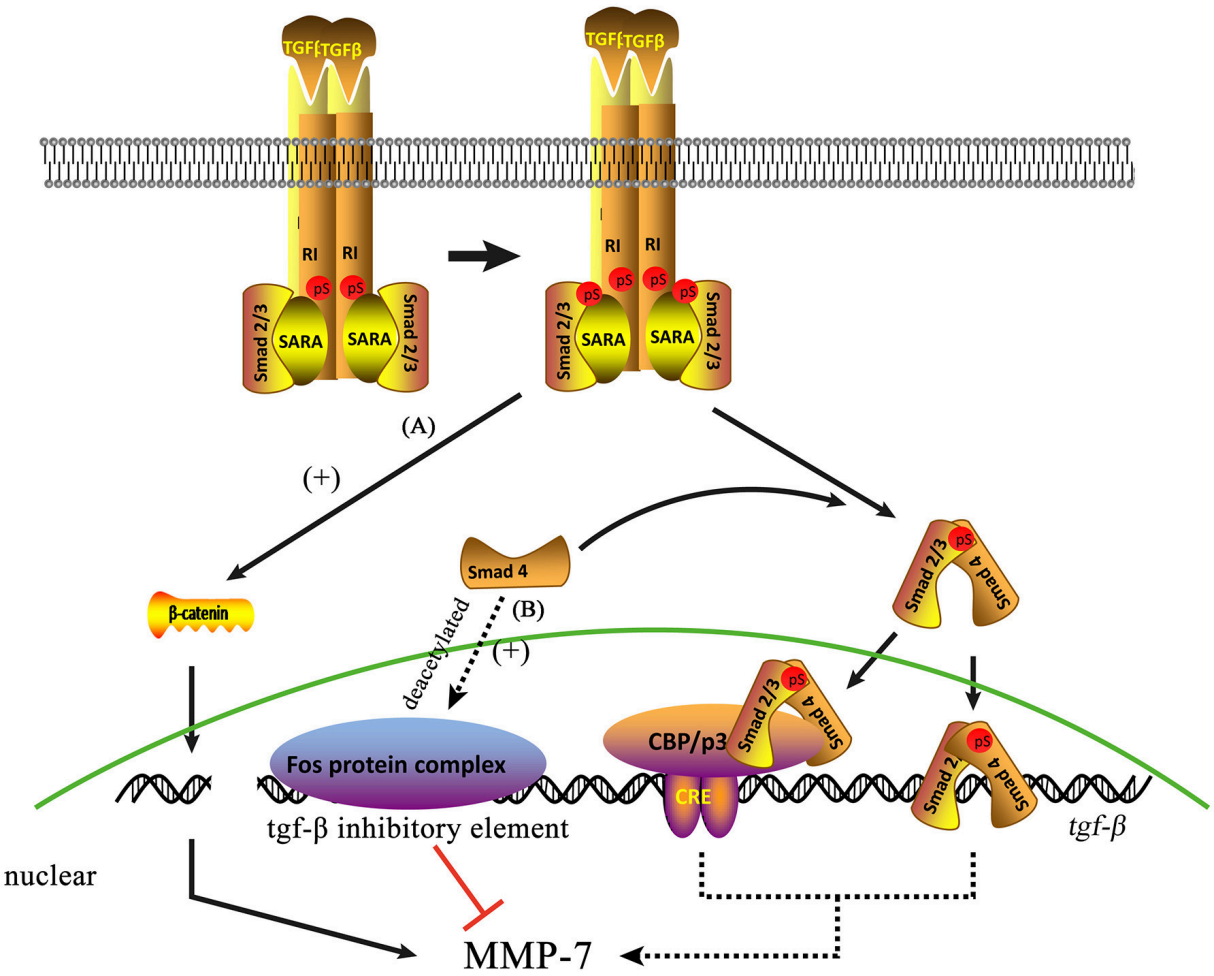
**Two molecular pathways may be involved in the mechanisms by which TGF-β regulates MMP-7 expression. (A)** TGF-β can induce β-catenin expression directly. **(B)** Smad4 deacetylation can suppress MMP-7 expression by promoting the binding of the Fos protein complex to the TGF-β inhibitory element, indicating that normal Smad4 can induce MMP-7 expression via TGF-β signaling.

Recent studies have revealed the existence of a close relationship between MMP-7 and TGF-β signaling (Figure 2). Li et al. (2017) observed that the expression of TGF-β1 and MMP-7 are upregulated in STZ-induced diabetic nephropathy rats but markedly decreased in diabetic nephropathy rats treated with sodium hydrosulfide (Li et al., 2017), which suggests regulation between TGF-β and MMP-7. MMP-7 is also closely related to TGF-β/Smad4 signaling (Xiao et al., 2016). Sirtuin 1 (SIRT1), a nicotinamide adenine dinucleotide (NAD+)-dependent class III deacetylase that can deacetylate both histone and non-histone proteins, has been shown to participate in numerous cellular processes by deacetylating specific substrates. In particular, SIRT1 has shown reno protective effects in several models of acute kidney injury (Fan et al., 2013) and chronic kidney diseases (Hasegawa et al., 2013). SIRT1 inhibits MMP-7 protein expression by deacetylating Smad4, a transcription factor for MMP-7 expression, whereas SIRT1 inhibition induces MMP-7 upregulation (Xiao et al., 2016). Simic et al. (2013) found that TGF-β regulates MMP-7 expression, whereas SIRT1 deacetylates Smad4 and represses MMP-7 activity. SIRT1 regulates MMP-7 expression and enzyme activity via Smad4 deacetylation. Moreover, Thiery et al. (2009) found that TGF-β-induced β-catenin is required for the synthesis of a-SMA, a marker of EMT, in renal proximal tubular cells. Moreover, β-catenin promotes EMT further by upregulating MMP-7(Zhou et al., 2013). However, TGF-β suppresses the expression of several MMPs by binding of the *c-fos* proto-oncogene product Fos protein to TGF-β inhibitory elements in the promoter regions of MMP genes (Kerr et al., 1990). McLennan et al. (2007) found that MMP-7 levels are reduced in streptozotocin-induced diabetic nephropathy rats via TGF-β-dependent mechanisms; thus, TGF-β induction may contribute to reductions in MMP-7 expression and ECM accumulation in diabetic nephropathy.

Interestingly, an MMP-7/syndecan-1/TGF-β1 autocrine loop leading to TGF-β1 production has been identified in hepatocellular carcinoma (Zeng et al., 2016). Further studies are needed to investigate whether a similar loop exists in renal fibrosis.

### MMP-7 and the ECM

Aberrant thickening of the glomerular basement membrane (GBM) and TBM, as well as excessive accumulation of mesangial matrices in renal fibrosis, is the result of an imbalance between the synthesis and degradation of ECM components. ECM deposition in renal structures, such as the glomerular capillary wall, arterioles, mesangium, and tubule-interstitial space, will ultimatelycause renal fibrosis (Duffield, 2014). The major ECM component perturbations that occur in the GBM include increases in collagen type IV (α 3 and α 4 chains), laminin and fibronectin expression (Hu et al., 2015). The molecules involved in increased ECM deposition in kidney fibrosis have been investigated extensively, especially MMP-7 (Xu et al., 2014).

MMP-7 can alter the micro-architecture of the glomerular apparatus and degrades collagen type IV, small proteoglycans, fibronectin, laminin, and entactin, all of which are components of the glomerular ECM (Tan and Liu, 2012). In addition, MMP-7 enhances the actions of growth factors. For instance, aberrant renal growth is preceded by a marked increase in the expression of intra-renal insulin-like growth factor (IGF)-I protein, which is trafficked into the kidney as a result of changes in IGF-binding protein (IGFBP) expression within renal tissues rather than enhancements in local IGF-I production (Flyvbjerg, 2000). Specifically, induction of IGFBP-1, 3, and 5 expression has been demonstrated in early renal hypertrophy in streptozotocin-treated diabetic rats (Park et al., 1998). Consequently, MMP-7 overproduction in kidney parenchyma may result in enhanced degradation of IGFBPs and the release of free bioactive IGFs, leading to expansion of the mesangium. MMP-7 has been shown to proteolytically degrade IGFBP-3 and IGFBP-5, and IGFBP cleavage by MMP-7 can liberate IGF-II (Miyamoto et al., 2004; Hemers et al., 2005; McCaig et al., 2006). Moreover, increased *Col1a2* and *Col3a1* expression are correlated with fibrotic changes in the kidney (Fragiadaki et al., 2011). A recent study reported that early MMP-7 upregulation increases *Col1a2* and *Col3a1* transcription primarily via PIK3, p38, ERK, Src, and PKA signaling, leading to subsequent collagen deposition in the kidney (Oelusarz et al., 2013). Taken together, these data suggest thatMMP-7 activity may contribute, either directly or indirectly, to GBM thickening and mesangial expansion, which precede the onset of kidney fibrotic lesions.

## Conclusion and perspective

MMP-7 plays an active role in renal fibrosis mainly through the following three pathways: EMT, TGF-β signaling and ECM deposition. These pathways interact with one another and contribute to the development of kidney fibrotic lesions. Several studies have suggested that MMP-7 is a potential target in renal fibrosis treatment. Resveratrol (RSV; trans-3,5,4′-trihydroxystilbene), a stilbene polyphenol from grapes, wine, mulberries, and peanuts, has been reported to have various pharmacological effects, including cardio protective effects in coronary heart disease, anti-inflammatory effects, and chemo preventive effects in cancer (Bhat et al., 2001; Aggarwal et al., 2004). RSV attenuates renal injury and fibrosis by inhibiting EMT, which is induced by upregulation of MMP-7 expression (Xiao et al., 2016). Moreover, compared with levels in normal subjects, patients with various kidney disorders have markedly elevated urinary MMP-7 levels, which are closely correlated with renal fibrosis scores in these patients (Zhou et al., 2016). In addition, among people with type 2 diabetes, urine, and serum MMP-7 levels are strongly associated with diabetic complications, namely, renal disease (Afkarian et al., 2015). Collectively, these results suggest that MMP-7 may not only be a potential target but also a noninvasive biomarker of kidney fibrosis.

However, a recent study investigating the potential correlation of urinary/serum MMPS/tissue inhibitors of metalloproteases (TIMPS) with subclinical progressive interstitial fibrosis and tubular atrophy (IF/TA) within the first 6 months post-renal transplant indicated that urinary MMP-7 was higher during subclinical tubulitis (*p* < 0.04) and that serum MMP-7 was barely correlated with subclinical IF/TA (Hirt-Minkowski et al., 2014). Thus, to deepen the understanding of the role of MMP-7 in the clinical treatment of kidney fibrosis, additional studies are needed to elucidate the molecular mechanisms by which MMP-7 contributes to the development of kidney fibrosis. These investigations will help researchers develop potential diagnostic biomarkers of and therapeutic targets for kidney fibrosis.

## Author contributions

XF and LY: Substantial contributions to the conception of the work, revising the article critically for important intellectual content, final approval of the submitted version, both agree to be accountable for all aspects of the work in ensuring that questions related to the accuracy or integrity of any part of the work are appropriately investigated and resolved. BK and CF: Drafting the article, final approval of the version to be published, agrees to be accountable for all aspects of the work in ensuring that questions related to the accuracy or integrity of any part of the work are appropriately investigated and resolved.

### Conflict of interest statement

The authors declare that the research was conducted in the absence of any commercial or financial relationships that could be construed as a potential conflict of interest.
