# Corrigendum: Matrix Metalloproteinase-7 and Kidney Fibrosis

**DOI:** 10.3389/fphys.2017.00192

**Published:** 2017-03-28

**Authors:** Ben Ke, Chuqiao Fan, Liping Yang, Xiangdong Fang

**Affiliations:** ^1^Department of Nephrology, The Third Hospital of NanchangNanchang, China; ^2^Nanchang University School of MedicineNanchang, China; ^3^Department of Breast Surgery, Jiangxi Cancer HospitalNanchang, China; ^4^Department of Nephrology, The Second Affiliated Hospital to Nanchang UniversityNanchang, China

**Keywords:** MMP-7, epithelial-mesenchymal transition, extracellular matrix, TGF-β

In this paper titled “Matrix Metalloproteinase-7 and Kidney Fibrosis,” there was a mistake in Ben Ke's department, which should be corrected. The correct work unit of Ben Ke should be Department of Nephrology, The Third Hospital of Nanchang, Nanchang, China. No other correction is needed as the text and figure legends are correct.

## Conflict of interest statement

The authors declare that the research was conducted in the absence of any commercial or financial relationships that could be construed as a potential conflict of interest.

